# Characterizing the CdSe nanodots in the vicinity of the monolayer covering range

**DOI:** 10.1039/c9ra09184j

**Published:** 2019-12-16

**Authors:** María J. Capitán, Jesús Álvarez, Sergio Puebla, Michael J. Spilsbury, Julio J. Conde, Beatriz H. Juárez, Roberto Otero

**Affiliations:** Instituto de Estructura de la Materia, CSIC c/Serrano 119 28006 Madrid Spain mj.capitan@csic.es; Física de Sistemas Crecidos con Baja Dimensionalidad, Universidad Autónoma de Madrid Unidad asociada al CSIC Spain; Dpto, Fisica Materia Condensada and the Condensed Matter Physics Center (IFIMAC), Instituto Nicolas Cabrera, UAM, Facultad de Ciencias CIII, Ctra, Colmenar Viejo km 14.5 28049 Madrid Spain; Dpto, de Química Física Aplicada, Facultad de Ciencias, UAM CIII, Ctra, Colmenar Viejo km 14.5 28049 Madrid Spain; IMDEA Nanoscience c\Faraday 9, Campus de Cantoblanco 28049 Madrid Spain

## Abstract

During the past decade, due to their large number of technological applications, a large number of research studies have been devoted to CdSe nanocrystal (NC) systems. Most of the studies of NC grown on substrates present in the literature correspond to a submonolayer coverage. However, interparticle interactions and, consequently, system morphology and its properties can change at higher coverage regime. We combine the X-ray diffraction technique at wide and small angle range with direct space AFM microscopy for the morphological characterization of samples in the monolayer vicinity. We conclude that the CdSe preserves its nanoparticle character and its pyramid shape. This nanoparticle character is also reflected in the CdSe Density Of States (DOS) measured by UPS. We have shown that the particle CdSe atoms are perfectly ordered. They form nanocrystals with a wurtzite structure, grown with an axial and lateral matching with the HOPG substrate lattice in a hexagonal arrangement up to the monolayer coverage, with a strong interaction with the substrate. Above the monolayer coverage this epitaxial match is looser, resulting in a 3D disorder growth.

## Abstract 2

We perform X-ray diffraction and AFM characterization of CdSe-nano-pyramids layers up to monolayer covering. The CdSe-particles are perfectly ordered growing with an axial and lateral matching with the HOPG substrate lattice in a hexagonal arrange.

## Graphical abstract

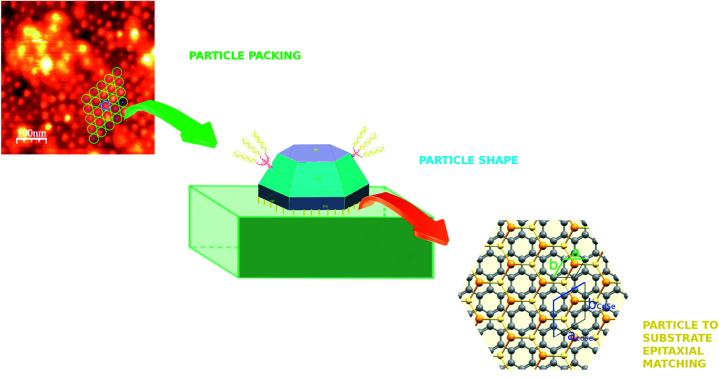

## Introduction

The binary CdSe semiconductor has a large field of technological applications, particularly when grown in nanodot size. This semiconductor type II–VI is considered an excellent candidate in optical devices technology, optical memories with high-density, transparent conductors, laser devices,^1^ photodetectors^2,3^ photoelectric sensors^4^ and solar cells.^5–8^ It can be also used as a biological label,^9^ and in spintronic devices.^10^ The CdSe is transparent in infrared radiation, thus it is sometimes used in the making of photoresistors and also, in thin layers, for instruments using infrared light. This material is also highly luminescent.

Over the past decades significant progress in the colloidal synthesis of size, shape, and composition-controlled semiconductor nanocrystals (NCs) has occurred.^11,12^ These colloidal complex structures are formed as a result of the interaction between different materials that lead the process. The critical issues that have to be addressed are: the inorganic particle–organic cover interface interactions; the nanodot to substrate interaction; and the inter-nanodots interactions, in particular those at high substrate covering range. Knowledge of these issues is essential in order to postulate reaction mechanisms that will allow full control and reproducibility over the preparation of complex structures.

In order to produce CdSe nanoparticles films, self-assembly approaches onto flat substrates have been reported, mainly by spin-coating and dip coating.^13–16^ An alternative methodology includes the self-assembly of NPs during the NP synthesis, an approach that produces CdSe NPs arrangements on graphitic substrates (flat surfaces such as HOPG or curved ones such as the surface of carbon nanotubes) with tunable coverage and excellent ordering, according to SEM and STM characterizations.^17–19^ This methodology produces CdSe NPs arrangements which have a strong resistance to being detached when immersed in non-polar organic solvents, suggesting a strong interaction between the CdSe system and the carbonaceous substrate.

In this study, different samples with different nanodots coverage were grown with the aim of determining if there would be any changes in the nanodots in the vicinity of the monolayer coverage due to the inter-particle interactions. Thus, here we mainly present the results of one sample with a coverage just above the monolayer coverage, in order to determine if the high concentration of particles induces changes in the particle shape, if they collapse or if they change their electronic properties. Most of the physical and chemical properties of such systems, such as light emission in quantum dots or catalytic properties of clusters, are intimately linked to their morphology. In this context, many techniques have been used to obtain accurate information on the nanometric scale. The Scanning-Probe Microscopies (SPM) in the two most popular variants: *i.e.* Atomic Force Microscopy (AFM) and Scanning Tunneling Microscopy (STM) have been widely used in conjunction with electron microscopy for the direct imaging of the sample surface. It is also possible to obtain morphological information in reciprocal space by using the small-angle X-ray scattering technique.^20,21^ Therefore, in the present study, we use direct space surface characterization by means of AFM combined with X-ray diffraction techniques based on the reciprocal space. X-ray diffraction has already been proven to be a powerful technique for the morphological characterization of deposited layers, multilayers and nanostructures such as quantum dots or supported islands. Principally due to the increasing use of synchrotron radiation, this technique has been extended to surface geometry, using the phenomenon of total external reflection of X-rays in the grazing-incidence geometry, which allows an enhanced surface signal.^22^ In the wide-angle scattering range, surface diffraction allows accurate information to be obtained on surface reconstructions, on surface relaxations and on atomic positions.^23,24^ The small-angle scattering range (grazing-incidence small-angle X-ray scattering (GISAXS)) gives information about the layer morphology and the lateral correlations, sizes and shapes of the clusters.^25–33^ Even though the SPM techniques can shed some insight into these types of surfaces, X-rays present several advantages: (i) they give averaged statistical information over the whole sample surface; (ii) they can be applied in various environments, ranging from ultrahigh vacuum to gas atmospheres, *in situ*, and in quasi real time when kinetics phenomena are involved; (iii) using the variable probed depth as a function of the incidence angle, X-rays offer the opportunity to characterize a range of phenomena from surface roughness to buried particles. By combining the advantages of synchrotron radiation and two-dimensional detectors with *in situ* sample preparation, the full potential of such a method can be exploited. Moreover, the GISAXS experiments can be performed with experiments involving X-ray diffraction at wide scattering angles (WAXS). In this configuration we will study the atomic structure of the clusters and their matching with the substrate.

However, apart from the topography or the tomography studies, the techniques known and employed to date cannot provide details about the interaction governing the interface or the electronic properties of the ensemble. It is well known that size-finite systems can induce special electronic features which can determine their electronic properties.^34^ There are a lack of UPS studies (a suitable experimental method for the determination of these DOS features) of the CdSe NCs in the literature. We have carried out in depth UPS studies and compared results with other theoretical studies present in the literature.^34,35^

## Experimental methods

### Sample synthesis

#### Chemicals

Cadmium oxide (99.998%), selenium powder (99.999%) and octadecylphosphonic acid (crystalline ODPA, 97%) were acquired from Alpha Aesar. Trioctylphosphine (TOP, 97%), was acquired in Sigma-Aldrich. Trioctylphosphine oxide (TOPO, 98%) toluene (99.5%) and 1,2-dichloroethane (DCE, 99.5%) were purchased from Merck.

#### Synthesis of CdSe NPs on HOPG surfaces

0.025 g (0.2 mmol) cadmium oxide, 0.200 g (0.6 mmol) octedecylphosphonic acid (ODPA), 2.9 g (7 mmol) trioctylphosphine oxide (TOPO) and a HOPG substrate are included in a rounded flask connected to a Schlenk line. With a slight agitation, the temperature is raised to 130 °C for 1 hour under vacuum to eliminate possible water content. The temperature is raised to 275 °C under N_2_ to form the Cd(ODPA)_2_ complex. Afterwards, the temperature is lowered to 80 °C and 4 μL (0.05 mmol) of 1,2-dichloroethane (DCE) is injected. The temperature is raised again to 265 °C to produce the nucleation of the NPs by injection of 0.43 mL of a selenium solution (0.4 mmol Se powder) previously dissolved in inert argon atmosphere in trioctylphosphine (TOP), immediately after injection the temperature is lowered to 255 °C to continue with the growth process for 21 hours.

#### Electronic characterization

The electronic properties were studied in an Ultra High Vacuum (UHV) system where different techniques are included; X-ray Photoelectron Spectroscopy (XPS) for the core level and chemical characterization using a Mg Kα line as photon source; and Ultra-violet Photoelectron Spectroscopy for the valence band measurements (UPS) by using an ultraviolet He discharge lamp. In both cases a hemispherical energy analyzer (LEYBOLD LHS10) was used for the photoelectron recording. The Mg K_α_ line (*hν* = 11 253.6 eV) was used for XPS and He-I (*hν* = 21.2 eV) and He-II (*hν* = 40.8 eV) lines for the UPS measurements. The pass energy of the analyzer was set to 50 eV for the XPS measurements to reach a resolution of 0.7 eV. The recorded XPS spectra was deconvoluted using a Richardson–Lucy algorithm^36^ in order to eliminate the MgK_α_ intrinsic line width and satellites.^37^ The iterative Richardson–Lucy procedure was applied until convergence, using as stopping criteria the appearance of a maximum in the Shannon-entropy. In such a way the information extracted is maximized, and the noise amplification is avoided.^36^ The deconvoluted XPS spectra are equivalent to spectra acquired with a monochromatic source. For the UPS, the pass energy was set to 5 eV, corresponding to a resolution of 0.07 eV. All the core levels energies in the XPS spectra are referred to a cleaved HOPG substrate used as reference. The energy of the UPS spectra are referred to the Fermi edge of a clean Cu(100) crystal.

#### Structural characterization

**Fig. 1:**
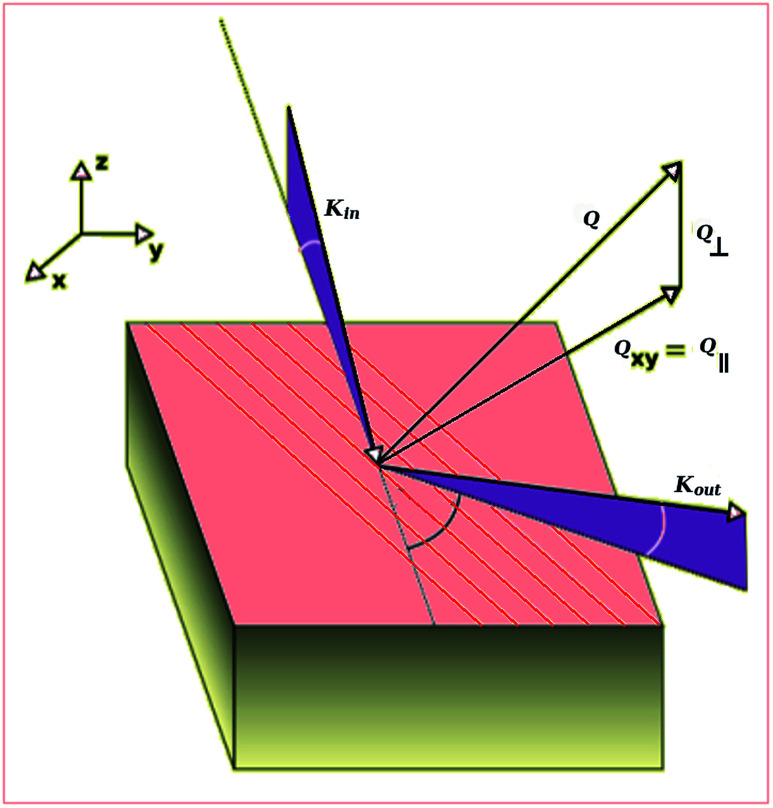
Sketch of the diffraction geometry with respect to the substrate. The parallel (*Q*_∥_) and perpendicular (*Q*_⊥_) momentum transfer magnitudes are shown with respect to the substrate surface. The *k*_in_ and *k*_out_ show the incident and outgoing beam.

The CdSe-nanodots film structure was characterized by means of surface X-rays diffraction at a fixed incidence angle of 2°. These measurements were performed at the P08 beamline (PETRA III storage ring) at Hasylab synchrotron at Desy using a wavelength of 1.397 Å. However, in the *θ*/*2θ* plots shown in this work, we have standardized the experimental diffraction patterns to the Cu K_α_ wavelength in order to be comparable to the diffraction patterns present in the literature. The experimental setup has a six-circle goniometer to allow a diffraction geometry, with fixed incoming beam angle, onto the crystal surface. A sketch of the geometry is shown in Fig. 1. The experimental resolution was defined by a pair of slits between the sample and the 0D-scintillator detector placed on a large detector arm (1.2 m of sample-detector distance). Reciprocal space maps were performed by scanning delta and gamma angles in the diffractometer (see Fig. 1) and recording the scattered signal with 0D-scintillator detector. The GISAXS obtained maps were analyzed using the BornAgain software using a 2D paracrystal model.^38^

Fig. 1 describes the different geometrical magnitudes used in the diffraction studies. In order to make a deeper study of our film morphology we made scans at different fixed outgoing beam angles (*γ*) with respect to the surface. In these scans the detector arm ran parallel to the substrate surface. This scattering geometry is widely used in surface X-ray diffraction. With this scattering geometry it is very common, in surface diffraction, to use a reciprocal space definition in which *H* and *K* vectors are parallel to the surface and *L* is perpendicular to the surface. With this definition scans in delta are mainly scans in *H* or *K* (parallel momentum transfer) and scans in gamma are mainly scans in *L* direction (perpendicular momentum transfer). Instead of the outgoing angle (*γ*) it is very common the use of other related magnitudes which are more lineal with the lattice distance; either the perpendicular momentum transfer parameter (*Q*_⊥_, being *Q*_⊥_ = 2 sin(*γ*/*λ*)) or, *L*_s_ which is the *Q*_⊥_ scaled by the substrate lattice parameter in the perpendicular to the surface direction (*L*_s_ = *Q*_⊥_*c*_HOPG(001)_ where s refers to substrate). Thus, *L*_s_ is a dimensionless magnitude which is inversely proportional to the relative size of the film lattice with respect to the substrate lattice in the perpendicular to the surface direction. In our case, the substrate is the HOPG (001), thus the *L*_s_ = 1 corresponds to a lattice equal to the *c* parameter of the graphite crystal (which is *c*_HOPG_ = 6.711 Å). On the other hand, the parallel momentum transfer is related to the angle of the outgoing beam within the substrate plane (*δ*). The total momentum transfer is the modulus of the parallel and perpendicular momentum transfer (*Q* = |(*Q*_∥_, Q_⊥_)|) and is related to the plane distance and the total diffraction angle (*θ*) by the Bragg law (*Q* = 1/*d* = 2 sin *θ*/*λ*).

The samples' morphology were also characterized by means of Atomic Force Microscopy (AFM) measures. The AFM experiments were performed in air using a Veeco Nanoscope VI multimode AFM working in tapping mode.

## Results and discussion

**Fig. 2:**
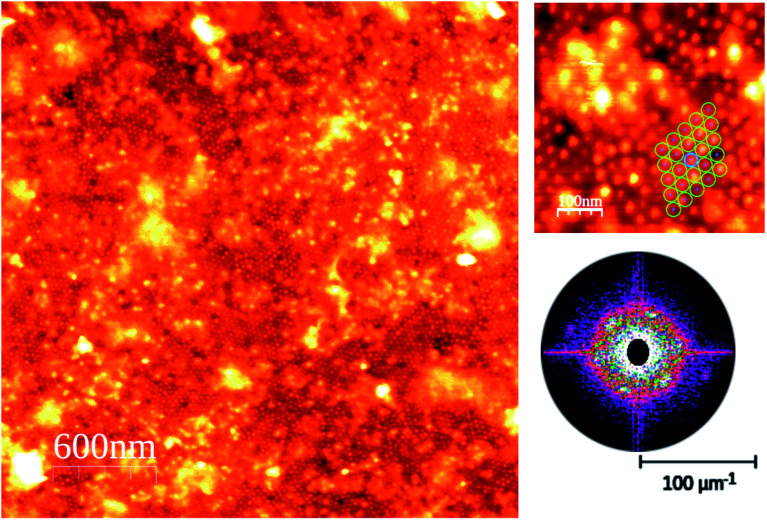
AFM images at different magnification scales of the sample just above the monolayer coverage. The lower right image is the Fast Fourier Transform (FFT) of the high magnification image. Observed is an initial CSe nanodots layer, well ordered and with a hexagonal order (shown in the low magnification scale image), together with other non-ordered and less homogeneous sized CdSe nanodots regions.

The AFM images of the CdSe nanodot sample at the monolayer coverage show two clear different regions in terms of ordering (see Fig. 2). The main region is an extended and very well-ordered region where it can be observed that the particle size is quite homogeneous within a hexagonal packing arrangement (see the high magnification image of Fig. 2). The AFM analysis indicates that the in-plane nanodots lattice is of 35 ± 3 nm, and the particles shape parameters are 6.3 ± 0.8 nm in width.^39^ The particle height is affected by the own technique's limits due to the difficulty in the tip penetration in very narrow and shape structures. The AFM shows that the nanoparticles are higher than 2.7 ± 0.8 nm height.

**Fig. 3:**
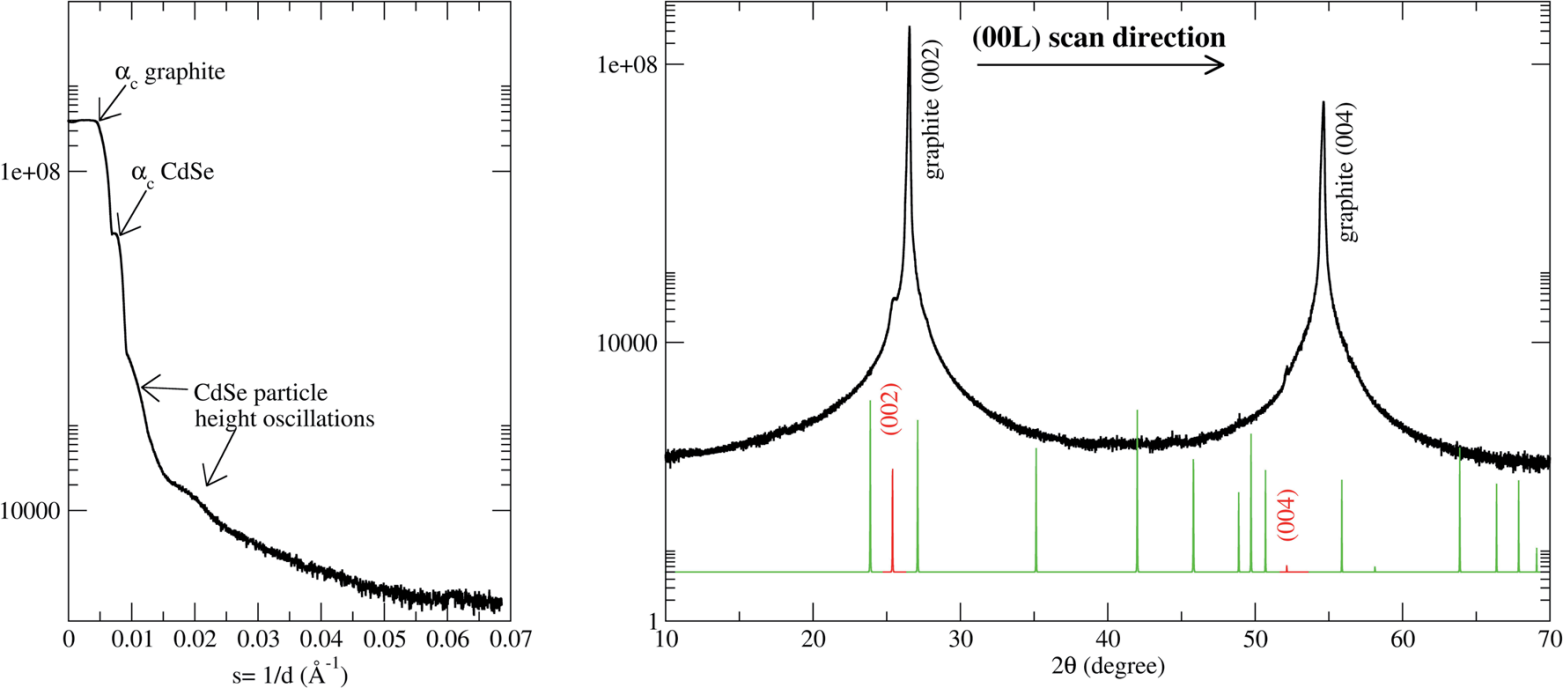
X-ray diffraction pattern measured along the perpendicular to the substrate direction (perpendicular momentum transfer direction). (Left panel) In the direct beam vicinity and, (right panel) wide scattering angles. The critical angles corresponding to the HOPG and CdSe are shown as well as the reflectivity oscillations corresponding to the CdSe NC. Spot lines show the Bragg peak positions for the hexagonal CdSe powder.^40^ The red lines correspond to the CdSe [002] Bragg peak family. These features are absent in the diffraction pattern of a freshly exfoliated HOPG substrate (not shown). In the latter case, the reflectivity curve only shows one critical angle and the 1/*Q*^2^ decay characteristics of a clean and flat surface. The CdSe peaks are absent in the wide-angle diffraction pattern.

The X-ray diffraction is able to provide information not only about the atomic order but also about the particle order and shape. The particle-related information is given at small outgoing angles (GISAXS) and the atom-related information at wide outgoing angles (WAXS). First, we shall concentrate our analysis on the scattering perpendicular to the surface. In such scattering geometry, we have sensitivity to the nanoparticle size in the perpendicular to the surface direction and atomic planes stack of the nanoclusters referenced to the HOPG substrate. Fig. 3 shows the 1D-pattern along the perpendicular to the substrate direction (*Q*_⊥_) at both small- and wide-angle scattering.

The small angle scattering shows information about the *z*-direction of the particle shape.^20–22^ Thus, there is an oscillation related to a particle height of 95 ± 5 Å, and also, two critical angles in the sample which are due to the very different electronic density of both the particles and substrate. The measured critical angles of 0.22 and 0.36 degrees are in good agreement with the tabulated values for the graphite and CdSe respectively and they correspond to refractive indexes of 7.4 × 10^−6^ and 1.9 × 10^−5^. The large difference implies a high substrate to nanodot contrast which is favorable for the X-ray study.

**Fig. 4:**
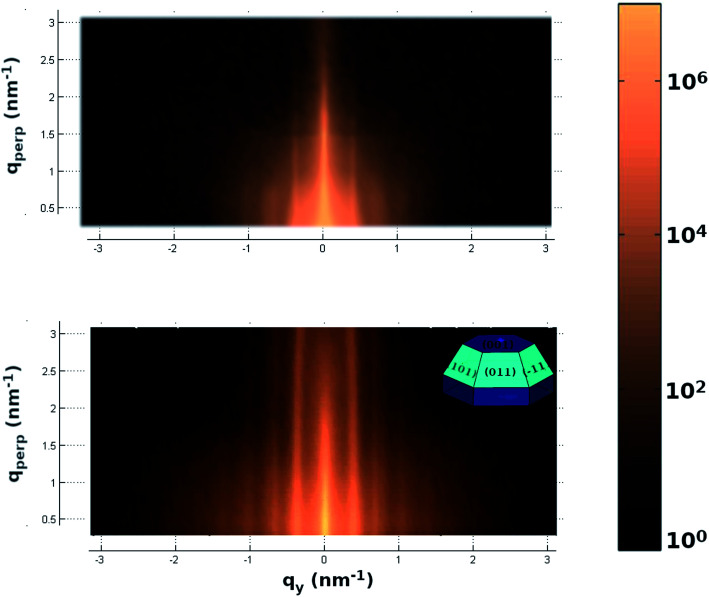
2-D small angle X-ray scattering pattern measured (upper panel) and simulated (lower panel).

In order to have not only information about the particles in the *z* direction but also about the lateral one, we measured the 2D small angle scattering (see upper panel of Fig. 4). This pattern shows the presence of peaks parallels to the perpendicular to the substrate direction (*Q*_⊥_). These peaks are related to the presence of a lateral inter-particle interference with a distance of 36.5 ± 1 nm with a hexagonal packing on the substrate surface. These results are in good agreement with the above-mentioned information given by the AFM. Furthermore, this 2D-pattern also provides information about each single particle shape. The pattern's fit using BornAgain software,^38^ shown in the bottom panel of Fig. 4, indicates a complex particle with a denser core (CdSe) and a less dense shell (the particle top cover). The particle shape given by the fit is a hexagonal truncated pyramid shape of 9 nm height, 12 nm lateral and 60 degrees in the core and a cover of 2 nm thick (sketch in Fig. 4 inset). This shape is in excellent agreement with the image given in the literature for low nanoparticles coverage on carbon nanotubes obtained by transmission electron tomography on graphitic surfaces.^18,41^

On the other hand, the 1-D pattern at wide-angle scattering shown in the right panel of Fig. 3 contains information about the atomic order of the sample. In the perpendicular to the substrate direction (*Q*_⊥_), the graphite [002] Bragg peaks family can clearly be observed (as it corresponds to the substrate orientation used) together with the CdSe [002] Bragg peaks family. This indicates that the atoms of the CdSe particles have a highly epitaxial orientation. The CdSe (001) planes are planes stacked parallel to the C (002) substrate surface in agreement with previous results for low coverage systems.^41,42^

**Fig. 5:**
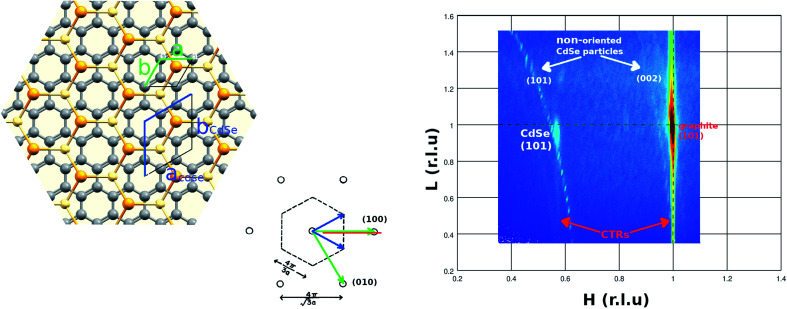
(Right panel) 2-D wide angle X-ray scattering pattern measured (right panel) along the HL-substrate lattice direction. (Left panel) comparative of the hexagonal HOPG substrate lattice with the hexagonal CdSe lattice.

In order to have information about the CdSe atomic order in the parallel to the substrate direction (*Q*_∥_) we have measured the 2D pattern at wide-angle scattering which is shown in Fig. 5. The scattering map corresponds to the graphite reciprocal space *HL*-plane (shown as a red line in the reciprocal space inset for reader clarity in figure). The measured pattern shows the graphite (101) Bragg peak with strain intensity along the perpendicular to the substrate direction corresponding to Crystal Truncation Rod (CTR) of a very flat graphite (002) surface. In addition to this, other peaks of the CdSe particles can be observed. The second in intensity is a single Bragg peak with CTR intensity at *H* = 0.568 and *L* = 0.957 which corresponds to CdSe (002). It is important to note that the presence of CTR signal on the Bragg peak of the nanoparticles reinforces the evidence that we are in the presence of a truncated pyramid. Considering that the graphite has a hexagonal lattice with *a* = 2.461 Å and *c* = 6.708 Å that gives a lattice of *a* = 4.33 Å and *c* = 7.01 Å which is in very good agreement with the experimental values given in the literature for CdSe hexagonal crystal.^40^ The atomic relationship between the graphite and the CdSe lattice is shown on the left panel of Fig. 5. Both lattice couplings are very good when the lattices are rotated 30 degrees one in respect to the other. This lattice match is in agreement with high-resolution transmission electron microscopy (HRTEM) studies of CdSe NPs on CNTs which have revealed the NPs shape and suggested an epitaxial matching between the [002] planes of CdSe wurtzite and the (001) planes of the outer graphene layer in the HOPG substrate.^42^ The CdSe nanodots are well crystallized, forming NCs. The CdSe (101) is observed just because block mosaics of different *x*, *y*-orientations are naturally present in the HOPG substrate.

Thus, by relating the WAXS and GISAXS results, we can give a complete description of the CdSe nanodots. The GISAXS fit indicates that the CdSe nanodot has a truncated pyramid shape with faces at an angle of 60 degrees with respect to the base, and, the WAXS data indicate that the CdSe nanodot base plane has an [001] atomic order. Thus, considering both, the pyramid faces seems to belong to the [101], [011], [111], [101], [011] and [111] family planes which form an angle of 62.03 degrees with the base in good agreement with the GISAXS fit.

In addition to this Bragg peak, the presence of ring-like intensity can also be observed. This intensity comes from non-oriented CdSe particles. Comparing samples with different coverage (not shown here) we have related this intensity to the second layer particles. The ring-like intensity decreases by decreasing the particle covering of the substrate disappearing below the monolayer nanodot covering. Thus, the lack of interaction of the second layer of nanodots with the well-ordered atoms of the substrate allow the CdSe particles to adopt a random orientation. We relate these non-oriented particles to the non-ordered region observed in the AFM images. Thus, this result suggests a strong and rigid C-graphite to CdSe interaction only for the first CdSe nanodots monolayer.

The XPS is capable of giving a description of the chemical environments of the atoms present in the sample. The measured XPS confirms the results shown in the literature for the CdSe nanoparticles^18^ with a light increase in the Cd intensity with respect to the theoretical to the Cd : Se 1 : 1 ratio. This means that the Cd atoms are segregated at the CdSe nanodots surface since the nanodot shell is richer in Cd than its core. The XPS edges of the C_1s_, O_1s_ and P_2p,_ which are the atoms implicated in the nanodot capping cover, were also measured, although they are not shown here because they are similar to those present in literature. Thus, the particle surface has a TOP capping cover.

**Fig. 6:**
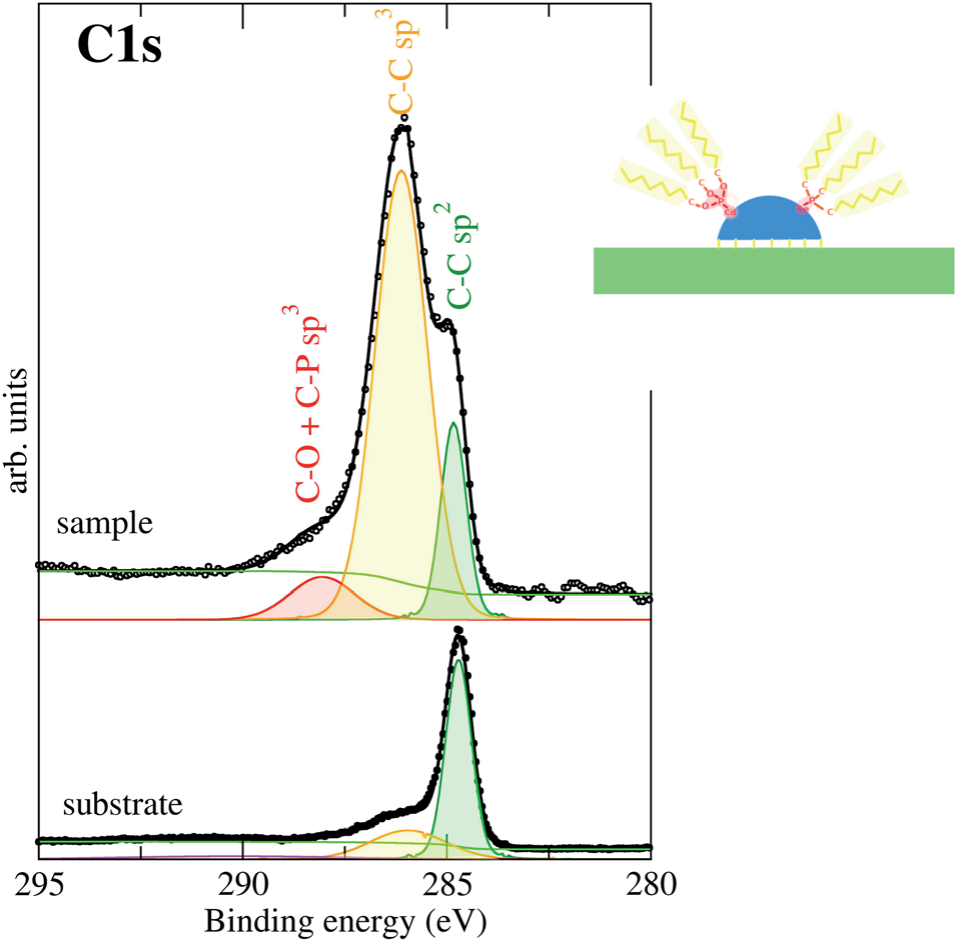
C 1s XPS spectra. Three components are observed corresponding to C–C sp^2^ bond (green), C–C sp^3^ bond (yellow) and C–O bond (red). The figure inset shows a sketch of the color used for the different components corresponding to different chemical environments of the elements.

The C 1s XPS signal requires special attention (Fig. 6). In the lower panel of Fig. 6 we can see the C 1s XPS peak corresponding to the clean freshly exfoliated HOPG surface. The two components of the peak correspond manly to C–C bonds with sp^2^ bonding hybridization (graphite structure) and a small peak at higher binding energy. It is known that this latter peak corresponds to C–C bonds with sp^3^ hybridization. Therefore, these sp^3^ bonds represent defects in the sp^2^ graphite structure probably coming from the structural defects (grain boundaries, exfoliation defects, *etc.*). For the CdSe covered sample, three different components are observed at the C 1s edge. They are assigned considering previous studies present in the literature.^43,44^ The lowest binding energy component is assigned to the C 1s C–C bonds with sp^2^ hybridization. As we have seen, this component is characteristic of the graphite, and therefore it is already present in the initial substrate.^44^ Following the color criteria shown in the inset above (inset of Fig. 6) this is shown in green. The highest C binding energy is related to C linked to P and/or O of the capping cover (shown in orange in Fig. 6). The third component, at intermediate binding energy (shown in yellow), is related to C bonds to C with sp^3^ hybridization and in the case of the CdSe covered sample, it should come mainly from the aliphatic C-chain. However, the intensity is higher than the addition of that initially present in the clean substrate (lower panel in Fig. 6) plus the trioctyl-chain of the capping cover. This excess of C-sp^3^ bonds present at the substrate can be at the base of the strong substrate-nanodot interaction. The strong interaction particle–substrate justifies the pyramidal shape of the particle because it maximizes the substrate to particle contact surface without losing its discrete behavior. The interaction is reinforced by bonds between substrate C sp^3^ defects with Cd or Se particle atoms. In other words, bonds between the HOPG substrate and the CdSe NCs atoms appear as C-sp^3^ defects in the ordered C-sp^2^ substrate network indicating that the substrate C-sp^2^ network breaks locally to bond to the NCs base pyramids. Previous studies on these hybrid systems have already pointed out that the interaction is triggered by the presence of chlorinated solvents during the synthesis, modifying the passivating ligand of the NPs that includes chlorine and promoting the interaction.^17,18^ Thus, these additional substrate C-sp^3^ defects should be promoted by the CdSe particle and in this particular case could be due to Cl atoms located in the NCs for the same given reasons. However, no traces of Cl or other elements were observed by XPS.

**Fig. 7:**
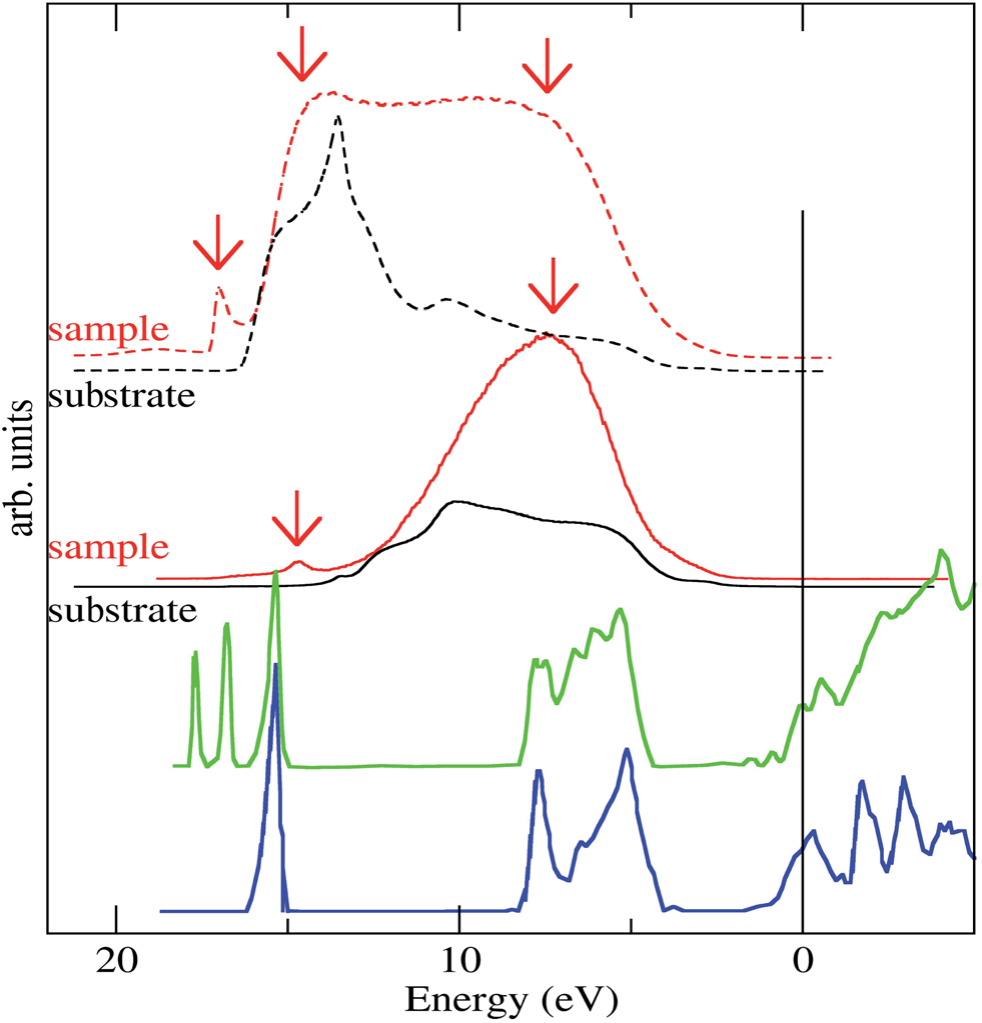
He-I UPS spectra of the CdSe-nanodot (red line) compared to the substrate spectra measured in the same conditions (black line). The upper lines correspond to the He-I UPS spectra measured polarizing the sample 5 eV. The spectra are compared to the calculated Density of State (DOS) calculated for CdSe bulk (blue line) and for a small size particle (green line).^35^

The UPS spectra gives information about the electronic structure of the systems. Fig. 7 shows the He-I spectra of the sample (red line) compared to the initial HOPG substrate (black line). A clear broad intensity at 7.3 eV and another small peak at 14.7 eV coming from the CdSe-nanodots can be observed. These intensity-signals can be compared to the Density of States (DOS) calculated for the CdSe shown in the literature.^34,35^ The DOS of bulk CdSe (blue line in Fig. 7) shows a broad peak mainly associated with the Se p-orbitals and a narrower peak at 6 eV bellow mainly associated with the Cd-d-orbitals. The absolute energy of the DOS calculation is limited by the zero position within the calculated band gap and the presence of the particle capping layer that could change the orbitals energy.^45^ This narrower peak energy is too close to the He-I energy cutoff and can be inferred by the presence of secondary electrons, either coming from the sample or from the electron analyzer. To overcome these difficulties we performed the UPS measures polarizing the sample −5 eV, which is a common procedure used to shift the spectra and avoid problems with extremely low kinetic energy electrons. In these experimental conditions we confirm the presence of energy coming from the CdSe particles at 7.5 eV and at 14.7 eV and also a new peak at 17 eV. Wang and Zunger.^35^ Applying their developed mesoscopic-scale pseudopotential method to CdSe quantum dots of different particles sizes has shown that this DOS Cd-level breaks into a few peaks in the quantum dot (green line in Fig. 7). The peak splitting is due to the finite particle size and comes from the surface atoms bonding states with one and two missing bonds, respectively. These peaks are very close to the photoemission cutoff. Moreover, the work function of the CdSe is higher than that observed in the graphite work function meaning that the photoemission cutoff in the CdSe particles should be higher than that observed in graphite.^46^ However, it is known that the chemisorption of chemical compounds on the CdSe can lower its work function by a surface dipole mechanism.^47^ In such a way, the observed UPS cut-off is compatible with the aforementioned situation. Therefore, the peak observed at 17 eV in the CdSe-nanodots sample could be ascribed, at first glance, to a secondary electron from the CdSe nanodots with a lower work function. However, we believe that this ascription is rather simple and that the origin this peak could evidence a more physically complex panorama and could be a manifestation of the effect described by Wang and Zunger.^35^ Indeed, the peak has not the typical intense and broad shape of secondary electrons. In fact, it is important to remember that secondary electrons arise from a physical phenomenon that came from the scattering of electrons in the solid loosing energy exciting plasmons or other inelastic processes. The probability of these processes is related to the inelastic mean free path. However, the inelastic mean free path for electrons at energies close to 5 eV (kinetic energy for these electrons) is around hundreds of Angstrom^48^ and much larger than the nanoparticle size. Therefore, it is rather possible that photoelectrons could escape from the CdSe nanoparticle without the generation secondary electrons. In this case, this additional peak in our measurement would indicate the limited nanoparticle size of this peak as described by Wang and Zunger.^35^ The presence of this additional peak in our measurement indicates the behavior as discrete particle behavior of our CdSe nanodot.

## Conclusions

We performed a deep structural characterization in real space (AFM) and reciprocal space (X-ray diffraction) of CdSe nanoparticles deposited on HOPG substrate in the complete monolayer coverage vicinity. We have shown that the particle CdSe atoms are perfectly ordered forming a nanocrystal with a wurtzite structure grown epitaxially on the HOPG substrate atoms owing to the fact that there is a strong C-substrate to CdSe-particles interaction in the first monolayer. Each individual particle shows a highly homogeneous pyramidal shape with the largest base face laying on the substrate surface. This arrangement derives from a strong NC–substrate interaction as depicted by a higher than usual presence of C sp^3^ signal in the XPS. This high intensity suggests that the sp^3^ excess signal came from surface defects confirming the aforementioned strong interaction in the bonding mechanism of the nano-particle with the substrate. There is not only an epitaxial matching between the [002] planes of CdSe wurtzite and the (001) planes of the outer graphite layer in the HOPG substrate but also a high degree of lateral order. Moreover, the GISAXs data confirms that the pyramids present a denser core when compared to the surface, which is indicative of the presence of the TOP particle cover. The pyramids show, up to the monolayer coverage, a hexagonal packing schema with a pyramid–pyramid distance of 36.5 nm. Above the monolayer coverage the CdSe NCs shows a non-ordered packing.

The valence band density of states measured by UPS is related to the CdSe density of states. We found evidences of finite size electronic structure effects, predicted in the literature in the UPS. The low energy part of the UPS spectra shows features that are compatible with the predicted finite size effects for CdSe nano-crystals with a similar size. This signal present in the UPS spectra is difficult to assign to a secondary electron onset, owing to the small size of the nano-particles involved in the presented experiments.

## Abbreviations

NC: Nanocrystals
GISAXS: Grazing-incidence small-angle X-ray scattering
TOP: Trioctylphosphine
TBAP: Tetrabutylammonium perchlorate
ODPA: Octadecylphosphonic acid
DCE: 1,2-Dichloroethane
TOPO: Trioctylphosphine oxide
UHV: Ultra high vacuum
XPS: X-ray photoelectron spectroscopy
UPS: Ultra-violet photoelectron spectroscopy
LEED: Low energy electron diffraction
HOPG: Highly oriented pyrolytic graphite
SPM: Scanning-probe microscopies
AFM: Atomic force microscopy
FFT: Fast fourier transform
CTR: Crystal truncation rod
DOS: Density of state

## Conflicts of interest

There are no conflicts to declare.

## Supplementary Material
